# Universal stress protein Rv2624c alters abundance of arginine and enhances intracellular survival by ATP binding in mycobacteria

**DOI:** 10.1038/srep35462

**Published:** 2016-10-20

**Authors:** Qiong Jia, Xinling Hu, Dawei Shi, Yan Zhang, Meihao Sun, Jianwei Wang, Kaixia Mi, Guofeng Zhu

**Affiliations:** 1 MOH Key Laboratory of Systems Biology of Pathogens, Institute of Pathogen Biology, Chinese Academy of Medical Sciences and Peking Union Medical College, Beijing 100730, China; 2 CAS Key Laboratory of Pathogenic Microbiology and Immunology, Institute of Microbiology, CAS, Beijing 100101, China; 3 The Research Institute of Forestry, Chinese Academy of Forestry, Beijing 100091, China; 4 Key Laboratory of the Ministry of Health for Research on Quality and Standardization of Biotech Products, National Institutes for Food and Drug Control, Beijing 100050, China; 5 Zhangjiakou Center for Adverse Drug Reaction and Drug Abuse, Hebei 075000, China; 6 College of Chemistry and Life Sciences, Zhejiang Normal University, Zhejiang 321004, China; 7 Shanghai Municipal Center for Disease Control & Prevention, Shanghai 200336, China

## Abstract

The universal stress protein family is a family of stress-induced proteins. Universal stress proteins affect latency and antibiotic resistance in mycobacteria. Here, we showed that *Mycobacterium smegmatis* overexpressing *M. tuberculosis* universal stress protein Rv2624c exhibits increased survival in human monocyte THP-1 cells. Transcriptome analysis suggested that Rv2624c affects histidine metabolism, and arginine and proline metabolism. LC-MS/MS analysis showed that Rv2624c affects the abundance of arginine, a modulator of both mycobacteria and infected THP-1 cells. Biochemical analysis showed that Rv2624c is a nucleotide-binding universal stress protein, and an Rv2624c mutant incapable of binding ATP abrogated the growth advantage in THP-1 cells. Rv2624c may therefore modulate metabolic pathways in an ATP-dependent manner, changing the abundance of arginine and thus increasing survival in THP-1 cells.

*Mycobacterium tuberculosis*, a successful ancient pathogen, causes the notorious infectious disease tuberculosis (TB). According to epidemiological data from the World Health Organization, two million deaths per year are caused by TB and approximately one-third of the world’s population is infected with *M. tuberculosis*^1^. Only 10% of infected individuals develop active pulmonary disease, the remaining 90%, while asymptomatic, carry latent bacilli. Immunosuppression, such as that caused by the human immunodeficiency virus, ageing and/or malnutrition, can lead to reactivation of latent bacilli, a major cause of adult tuberculosis^2^. Mechanisms underlying latency and reactivation have yet to be completely defined, and characterization of mycobacterial proteins that play important roles in latency and reactivation may provide insights for designing new therapeutic strategies to treat latent infections.

Universal stress proteins, characterized by the presence of a conserved G2 × G9 × GS motif, are present in organisms from bacteria to eukaryotes (but not in animals) and are highly induced under certain stresses, such as heat shock, superoxide-generating agents and heavy metals^3^. *Escherichia coli* UspA was the first universal stress protein identified^4^ and has been shown to respond to a large variety of stress conditions^5^,^6^,^7^. Deletion of *usp*A in *E. coli* arrests growth at the stationary phase^6^. Usually there are multiple universal stress proteins in a given species: *E. coli*, for example, possesses six paralogous genes — *usp*A, *usp*C, *usp*D, *usp*E, *usp*F and *usp*G. Deletion of individual *usp* genes has shown that they have different roles in response to cellular stresses^8^. Like *E. coli*, multiple universal stress proteins have been predicted in the mycobacteria, e.g., *M. tuberculosis* (NC_000962.3) is predicted to possess 10 universal stress proteins^9^ which are highly induced when triggered by different stimuli or environmental conditions^10^. Of these, six, including Rv2624c, are predicted to be in the DosR/Rv3133c regulon^11^,^12^, suggesting that universal stress proteins modulate latent infection. The *M. tuberculosis* universal stress protein Rv2623 is known to be involved in mycobacterial persistence^13^, and Rv1996 in isoniazid susceptibility^14^. The exact physiological roles of the other mycobacterial universal stress proteins, however, are unknown.

A microarray study has shown that *rv2624c* mRNA is highly induced by nitrosative stress, a host innate immune system-induced natural stressor against infection^15^. In addition, Rv2624c has been shown to be an immunogenic protein, the antibody against Rv2624c being detected in serum from TB patients serum^16^. These studies suggest that Rv2624c affects the infected host cell immune response. Knockout of a *usp* gene is compensated for by other universal stress proteins in mycobacteria^17^, while overexpression of a universal stress protein provides information about its function as overexpression increases its effect over that of other universal stress proteins. For example, overexpression of *rv1996* increased isoniazid susceptibility^14^. Overexpression of universal stress protein *rv2623* arrested growth of both *M. smegmatis* and *M. tuberculosis*^13^. In this study, we overexpressed *rv2624c* in mycobacteria and investigated the physiological functions of Rv2624c. We showed that Rv2624c increases the survival of mycobacteria in hypoxia and bacillary-infected THP-1 cells. Transcriptome analyses suggested that Rv2624c affects histidine metabolism and arginine and proline metabolism. Furthermore, LC-MS/MS analysis showed that Rv2624c affects the abundance of arginine, a modulator of both mycobacteria and infected THP-1 cells. In addition, biochemical analysis showed that Rv2624c is a nucleotide-binding universal stress protein. The growth advantage in THP-1 cells is ATP dependent, as an Rv2624c mutant incapable of ATP-binding abrogated the growth advantage of *M. smegmatis* mc^2^155 cells overexpressing Rv2624c.

## Results and Discussion

### Overexpression of Rv2624c increases mycobacterial survival in the Wayne mycobacterial model of latency

**Figure 1:**
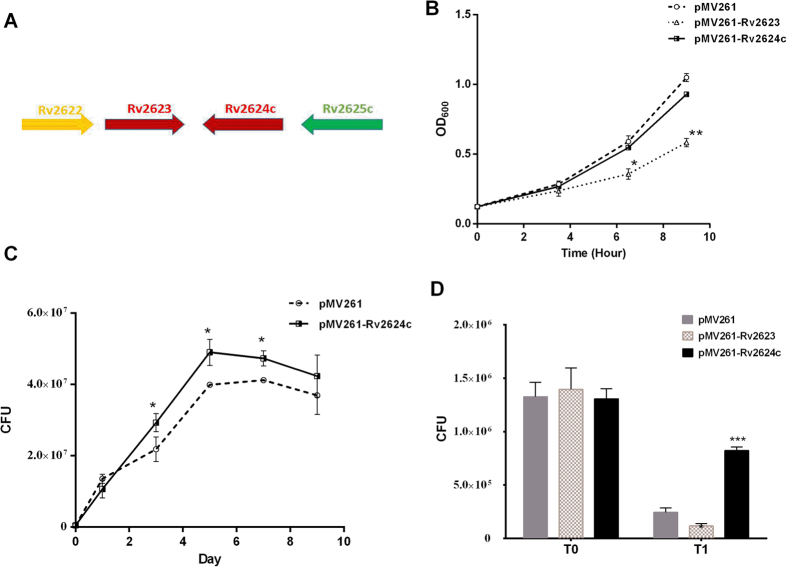
Overexpression of *rv2624c* in *M. smegmatis* increases survival in the Wayne hypoxia model. (**A**) Genetic organization of the *rv2624c* gene locus. (**B**) Growth curve of pMV261/mc^2^155, pMV261-Rv2623/mc^2^155 and pMV261-Rv2624c/mc^2^155 cells in 7H9 medium. *p < 0.05, **p < 0.01. (**C**) Overexpression of Rv2624c increased survival under hypoxia. Growth curves of pMV261/mc^2^155 and pMV261-Rv2624c/mc^2^155 under hypoxia. The results shown are representative of three independent experiments. *p < 0.05, **p < 0.01. (**D**) Overexpression of *rv2624c* in *M. smegmatis* increases survival in human monocyte THP-1 cells. *p < 0.05, ***p < 0.001.

In the *M. tuberculosis* genomic context, *rv2624*c neighbors *rv2623* but is in the opposite orientation (Fig. 1A)^17^. Rv2623 is the first mycobacterial universal stress protein identified that has been shown to be involved in *M. tuberculosis* persistence *in vivo* and to regulate mycobacterial growth *in vitro*^13^. Like Rv2623, Rv2624c has been shown to be highly induced under NO and hypoxia, and within infected macrophages^9^,^11^,^12^. However, the function of Rv2624c has not been reported as yet.

We first examined whether overexpression of *rv2624c* affects mycobacterial growth. Consistent with a previous study showing that Rv2623 overexpression arrests growth^11^, the pMV261-Rv2623/mc^2^155 strain grew slowly compared with the control *M. smegmatis* mc^2^155 strain harboring an empty vector (pMV261/mc^2^155) (Fig. 1B). In contrast, under the experimental conditions used here, growth of pMV261-Rv2624c/mc^2^155 was comparable with pMV261/mc^2^155, indicating that overexpression of *rv2624c* did not affect mycobacterial growth (Fig. 1B).

As universal stress proteins play roles in latent infection, we investigated the effects of Rv2624c in the Wayne mycobacterial latency model in which mycobacterial cultures are grown under gradual oxygen concentration depletion to force bacilli to enter a physiologically latent state. Bacteria (OD_600_ of 0.01) were seeded into vials with a headspace: liquid ratio of 1:2 supplemented with the O_2_ probe methylene blue, sealed and incubated with slow stirring (Fig. 1C). No difference in viability was detected on the first day after inoculation (Fig. 1C). Differences in the viability of pMV261-Rv2624c/mc^2^155 under hypoxia compared to pMV261/mc^2^155 were observed on days 3, 5, and 7 after inoculation (Fig. 1C). These results suggest that Rv2624c affects viability under hypoxic conditions.

### Overexpression of *rv2624c* in *M. smegmatis* increases survival in human monocyte THP-1 cells

Formation of granulomas is a hallmark of the host response to infection with *M. tuberculosis.* The hypoxic microenvironment within granulomas is an important factor leading to mycobacterial latency^18^,^19^,^20^,^21^,^22^. Rv2624c activity is required for survival under hypoxia, suggesting that latency gene *rv2624c* likely modulates the host immune system. In addition, studies have shown that Rv2624c is an immunogenic protein^16^,^23^. Macrophages are central innate immune cells that play a key role in the host response against pathogens. Using a macrophage-killing assay, we evaluated the intracellular survival of mycobacterial strains in the macrophage-like cell line THP-1 after a synchronized infection (Fig. 1D). After extensive washing, cells were lysed and spread on 7H10 medium (T_0_) to determine how many bacilli had infected the cells. As shown in Fig. 1D, the initial numbers of pMV261/mc^2^155 bacteria infecting THP1 cells was comparable to that of pMV261-Rv2624c/mc^2^155, indicating there was no statistical difference between strains in their entry into host cells. At 4 h post-infection, the number of pMV261-Rv2624c/mc^2^155 CFUs (8.23 × 10^5^ ± 2.4 × 10^4^), was significantly higher than that of pMV261/mc^2^155 CFUs (2.4 × 10^5^ ± 3 × 10^4^). This result suggests that overexpression of *rv2624c* increased mycobacterial survival in THP-1 host cells. In contrast, overexpression of *Rv2623* attenuated growth in THP-1 cells (1.2 × 10^5^ ± 1.5 × 10^4^ CFUs) (Fig. 1D). Those results showed that overexpression of *rv2623* decreased survival and overexpression of *rv2624c* increased survival in human monocyte THP-1 cells.

### mc^2^155 and pMV261-Rv2624c/mc^2^155 cells have distinct metabolic patterns

**Figure 2:**
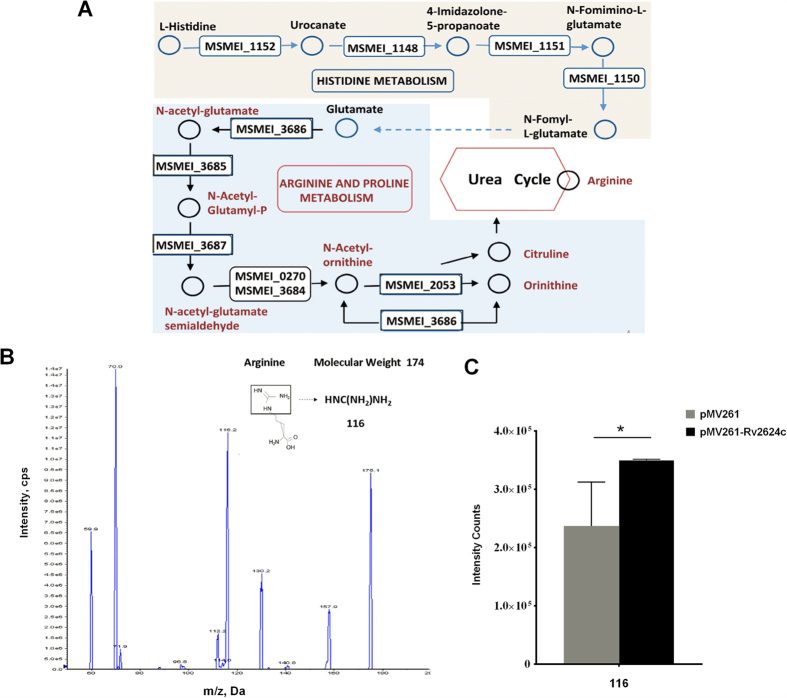
Overview of the differential expression profiles of pMV261/mc^2^155 and pMV261-Rv2624c/mc^2^155 cells. (**A**) *M. smegmatis* metabolic pathways in which DEGs are enriched. KEGG-User Data Mapping assigned DEGs to “histidine metabolism” and “arginine and proline metabolism”. (**B**) Mass spectrometry of arginine (m/z 175, 116, 70). (**C**) Ion intensities of fragment ions of arginine. *p < 0.05.

Transcriptional reprogramming plays important roles in bacterial responses to various stressors. To elucidate why pMV261-Rv2624c/mc^2^155 cells have a growth advantage over pMV261/mc^2^155 cells under the tested conditions (hypoxia and infection of THP-1 host cells), we examined changes in mRNA expression using RNA sequencing. Comparing the RNA of pMV261-Rv2624c/mc^2^155 cells to that of pMV261/mc^2^155 cells showed modest expression differences. Two hundred and eighty-six were upregulated more than 2-fold and 33 genes were upregulated more than 4-fold, respectively (*p* < 0.01), while 11 genes were downregulated. As different genes cooperate to perform their biological functions, pathway-based analysis gives insight into the Differential Expressed Genes (DEGs) involved in biological functions. KEGG Pathway Analysis^24^ showed that “arginine and proline metabolism” (Q = 0.004), “tryptophan metabolism” (Q = 0.013) and “histidine metabolism” (Q = 0.013) pathways are significantly enriched in DEGs compared to the whole *M. smegmatis* genome. As listed in Table 1, 15 DEGs are involved in histidine metabolism (pathway ID ko00380) and 19 upregulated genes are involved in arginine and proline metabolism. Interestingly, using KEGG-User Data Mapping (Fig. 2A), four genes were shown to be involved in “histidine metabolism”, which includes the amino acid synthesis pathway from histidine to glutamate, and seven genes were shown to be involved in “arginine and proline metabolism”, an arginine synthesis pathway. Arginine is an important modulator of infected macrophages and the pathogens which have infected them^25^,^26^, and arginine as an adjuvant for chemotherapy for active tuberculosis improves clinical outcomes^27^. There are many catabolic pathways for pathogens to degrade arginine, including the arginine deiminase pathway and the arginase pathway, the former supporting growth under anaerobic conditions and the latter supporting growth under aerobic conditions. Therefore, arginine metabolism is essential for both the host and the pathogen, and competition for arginine and thus may determine the outcome of infection^28^. Some pathogens have been shown to alter their arginine-dependent metabolic activities when infecting host cells. For example, *M. marinum* induces *argS*, an arginine metabolism gene, when it is inside host cells^29^. Results from our transcriptome analysis suggest that differences in the abundance of arginine may be important in mycobacterial survival within infected macrophages.

### Overexpression of Rv2624c increases the abundance of arginine

To examine whether Rv2624c affects arginine synthesis, we measured the abundance of arginine in pMV261 and pMV261-Rv2624c bacterial cells using mass spectrometry. As metabolites are easily altered by simple experimental procedures such as centrifugation, we used the filter-culture approach described by de Carvalho *et al.*^30^ to extract metabolites without significantly altering metabolite profiles. We then compared the abundance of arginine in pMV261/mc^2^155 and pMV261-Rv2624c/mc^2^155 using LC-MS/MS. The structure of arginine and its two major fragments at *m*/*z* 70 and 116 is shown in Fig. 2B. The abundance of arginine was statistically higher in pMV261-Rv2624c/mc^2^155 than in pMV261 (Fig. 2C).

### Rv2624c is a nucleotide-binding universal stress protein

**Figure 3:**
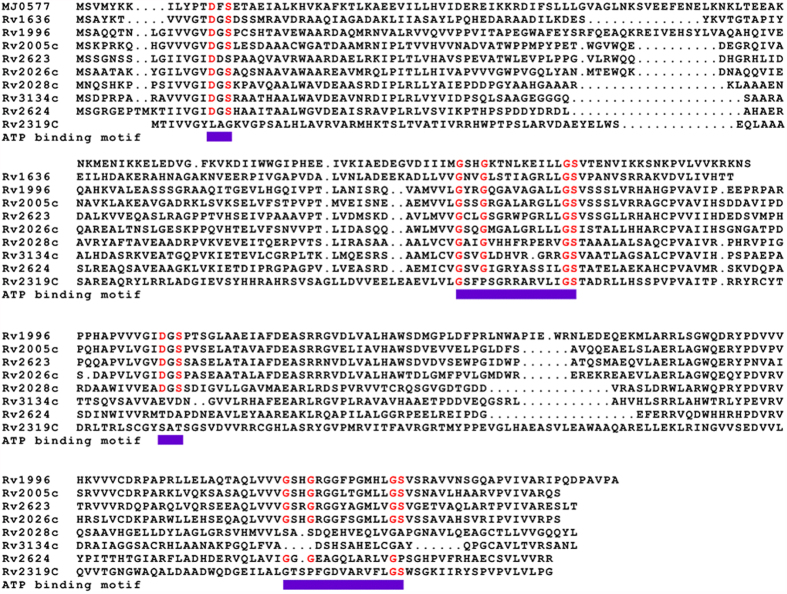
Rv2624c sequence analysis. Alignment of the universal stress protein domain of Rv2624c with other universal stress protein family proteins. The compared universal stress protein sequences are from DNAMAN V6 software. The predicted ATP-binding sites are indicated below the sequence.

**Figure 4:**
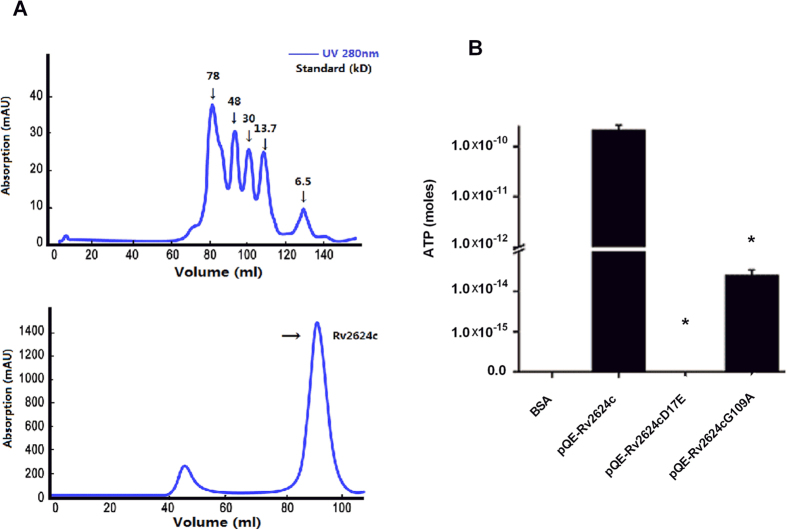
Rv2624c is a nucleotide-binding universal stress protein. (**A**) Gel filtration analysis of native His_6_-Rv2624c. The upper panel indicates the retention time of the corresponding standard proteins. The lower panel indicates the retention time of His_6_-Rv2624c. (**B**) The ATP-binding capacity of mutant Rv2624c was compared with that of wild-type Rv2624c. *p < 0.05.

**Figure 5:**
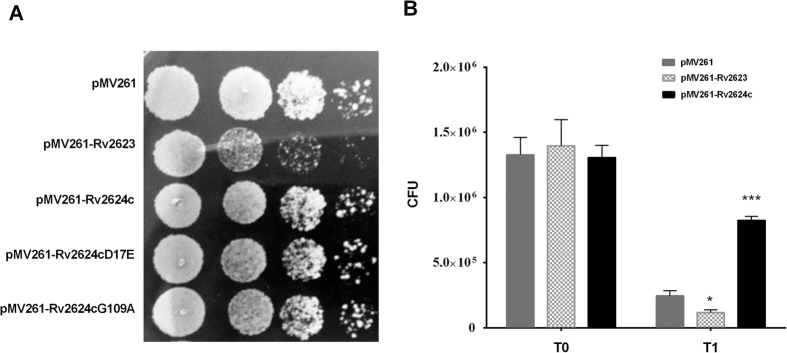
ATP binding by Rv2624c is required for its ability to increase survival in infected THP-1 cells. (**A**) The ATP-binding deficiencies of Rv2624c mutants did not influence growth on 7H10 medium. The mycobacterial strains indicated were serially diluted and were spotted onto solid 7H10 medium with 10% OADC. Pictures were taken after 3 d of incubation at 37 °C. (**B**) ATP-binding ability affects the survival of mycobacterial strains in THP-1 cells. Rv2624c mutant D17E with abrogated ATP-binding ability attenuated survival in THP-1 cells. The viability of Rv2624c mutant G109A, which had reduced ATP-binding ability, was comparable with that of wild-type Rv2624c. All results presented are representative of three independent experiments. *p* < 0.001.

Universal stress proteins are an ancient protein family, present in organisms from bacteria to plants, but absent in animals. Based on their ATP-binding ability, universal stress proteins have been assigned to two subclasses: one whose members do not bind nucleotides and the other whose members bind nucleotides^31^. A previous study has shown that mycobacterial universal stress protein Rv2623 is an ATP-binding protein and that the ATP binding of Rv2623 regulates bacillary growth^13^. Alignment of universal stress proteins, including the well-studied Rv2623, predicted that Rv2624c is an ATP-binding protein (Fig. 3), suggesting Rv2624c might have ATP-dependent biological functions. In addition, transcriptome analysis suggested that overexpression of Rv2624c affects the mycobacterial histidine, and arginine and proline metabolic pathways, which both use ATP. To determine if Rv2624c has ATP-dependent biological functions, we investigated the biochemical characteristics of Rv2624c protein to elucidate whether it affects growth in infected host cells in an ATP-dependent manner. *M. tuberculosis* Rv2624c was cloned and expressed in *E. coli.* SDS-PAGE analysis of purified His_6_-tagged Rv2624c protein showed a single band around the predicted molecular mass of ~30 kDa that was confirmed by immunoblotting to be Rv2624c (data not shown). Gel filtration analysis of native His_6_-Rv2624c proteins showed that the majority of the purified Rv2624c had a molecular mass of ~30 kDa (Fig. 4A), indicating that Rv2624c is a monomer under native conditions. Similar to Rv2623^13^, purified Rv2624c proteins from *E. coli* bound tightly to ATP and ADP after extensive purification steps (Figure S1), indicating that Rv2624c is a nucleotide-binding protein. To gain insight into the function of ATP binding, the amino acid sequence of Rv2624c was compared with that of Rv2623 (Fig. 3). In a previous study on Rv2623, G117 and D15 were identified as key amino acids for ATP binding^13^. When we mutated the corresponding amino acids in Rv2624c (D17, equivalent to D15 in Rv2623, and G109, equivalent to G117 in Rv2623) to E and A, HPLC analysis of nucleotides extracted from Rv2624c_D17E_ and Rv2624c_G109A_ showed that both mutations had abrogated the protein’s ATP-binding ability (Fig. 4B). We next compared the growth of mycobacterial strains overexpressing Rv2624c, Rv2624c_D17E_ and Rv2624c_G109A_. When measuring the OD_600_ values, no growth differences were detected among the strains (data not shown). Consistent with this result, the size of mycobacterial colonies was similar (Fig. 5A). In contrast, strains overexpressing Rv2623 showed arrested growth^13^ and formed small colonies (Fig. 5A). We also examined the survival percentage of these mycobacterial strains in infected macrophage cells. In contrast to their growth on 7H10 medium, we observed statistically significant differences in growth between mc^2^155 cells overexpressing Rv2624c and mc^2^155 cells overexpressing Rv2624c_D17E_ (p < 0.001), but not mc^2^155 overexpressing Rv2624c_G109A_. As in the macrophage killing assay studies, the percentage of *M. smegmatis* strains overexpressing mutant Rv2624c_D17E_ that survived in infected THP-1 cells was reduced (Fig. 5B). Growth in THP-1 cells infected with cells expressing the G109A mutant was comparable with that of cells expressing wild-type Rv2624c (Fig. 5B). The distinct effects exhibited by the wild type, and the G109A and D17E mutants defective in ATP binding suggest a correlation between survival in infected host cells (THP-1 cells) and ATP binding. Studies on mycobacterial universal stress proteins appear to indicate that they are involved in energy-related biological functions, e.g. Rv2623 regulates bacillary growth by ATP binding^13^, and Rv1996 regulates NAD/NADH-related isoniazid susceptibility^14^. Rv1636, another mycobacterial universal stress protein, has also recently been shown to bind ATP and is predicted to function in energy (i.e., ATP)-dependent pathways^32^. Based on the results of this study, and our understanding of the literature, we suggest that mycobacterial universal stress proteins regulate different energy-related pathways and are thus potential drug targets for antibiotics selection.

## Conclusions

We have shown that Rv2624c overexpression in mycobacteria increases their survival in the Wayne mycobacterial latency model and in human monocyte THP-1 cells. Transcriptome analysis suggests that Rv2624c affects histidine metabolism, and arginine and proline metabolism. Further studies using LC-MS/MS showed that Rv2624c modulated the abundance of arginine, a modulator of both mycobacteria and infected macrophages. In addition, biochemical characterization showed that Rv2624c is a nucleotide-binding universal stress protein and modulates metabolic pathways in an ATP-dependent manner, changing the abundance of arginine and thus increasing survival in THP-1 cells.

## Materials and Methods

### Reagents and growth media

Mycobacterial strains were grown in liquid Middlebrook 7H9 medium (Becton Dickinson, Sparks, MD, USA) containing 10% OADC supplement (oleic acid, albumin, dextrose, catalase; Becton Dickinson, Sparks, MD, USA) and 0.05% Tween 80 (Sigma, St. Louis, MO, USA), or on solid 7H10 medium (Becton Dickinson, Sparks, MD, USA) containing 10% OADC supplement. When needed, kanamycin (50 mg/L; AMRESCO, Shanghai, China) was added to the medium. *Escherichia coli* strains were grown in Luria-Bertani medium. Kanamycin (50 mg/L) and ampicillin (100 mg/L) were used as needed.

### Construction of the *rv2624c*-overexpressing mycobacterial strain and *E. coli* expression strain

The full-length *rv2624c* gene was amplified from *M. tuberculosis* H37Rv genomic DNA using sense (Rv2624c-F) and antisense primers (Rv2624c-R) (Table S1). The PCR reaction conditions were 98 °C for 30 s, followed by 30 cycles of 98 °C for 10 s, 60 °C for 30 s and 72 °C for 30 s, and then 72 °C for 10 min. The PCR-amplified fragments were purified, digested and cloned into *E. coli* expression vector pQE80L (Qiagen, Frankfurt, Hesse-Darmstadt, GER), named pQE-Rv2624c for expression of His_6_-Rv2624c, and mycobacterial vector pMV261, named pMV261-Rv2624c. pQE-Rv2624c was transformed into BL21 (DE3) for expression of in-frame 6xHis-tag recombinant Rv2624c and pMV261-Rv2624c was transformed into *M. smegmatis* wild-type strain mc^2^155 for overexpression of Rv2624c in mycobacteria.

### Establishment of the Wayne hypoxia latency model in mycobacteria

*M. smegmatis* strains were cultured under relatively hypoxic conditions as previously described^12^. Briefly, cultures of mc^2^155 strains were grown in 7H9 media at 37 °C to an OD_600_ of 1.0. Cultures were then inoculated at a concentration of 1 × 10^6^ CFU/ml (initial OD_600_ of 0.02) in tightly sealed anaerobic bottles. The cultures were set up to achieve a headspace ratio of 0.5 as defined by the Wayne Model^19^ and methylene blue (1.5 mg/L) was used as an indicator of oxygen concentration. All cultures were set up in triplicate.

### Purification of recombinant Rv2624c from *E. coli*

Recombinant Rv2624c was induced by incubation with 1 mM isopropyl β-D-1-thiogalactopyranoside (IPTG) at 37 °C for 3 h. Cells were harvested and resuspended in lysis buffer (20 mM Tris-HCl pH 8.0, 200 mM NaCl, 15 mM imidazole). The lysis buffer containing expressed recombinant Rv2624c protein was lysed by sonication, and then centrifuged at 12,000 *g* for 30 min at 4 °C for clearance. The supernatant was incubated with Ni-NTA agarose (Qiagen) and the agarose with bound Rv2624c proteins was washed using wash buffer (20 mM Tris-HCl pH 8.0, 200 mM NaCl, 50 mM imidazole). The Rv2624c protein was eluted with elution buffer (20 mM Tris-HCl, 200 mM NaCl, 300 mM imidazole) and the enriched Rv2624c protein was passed through a Superdex 200 10/300 GL column (GE Healthcare, Fairfield, Connecticut, USA) in a buffer containing 200 mM NaCl and 20 mM Tris-HCl, pH 7.0, for further purification. Purified Rv2624c proteins were concentrated and stored at −80 °C for further experiments. Mutant Rv2624c proteins were purified using the same protocol as described above for Rv2624c protein purification.

### Filter-culture approach for extract of metabolites

*M. smegmatis* strains (10 ml culture, OD_600_ of 0.6–0.8) was inoculated onto 10-mm 0.22 μm nitrocellulose filters under vacuum filtration. Corresponding *M. smegmatis*-laden filters were then placed on top of 7H10 media and incubated overnight at 37 °C. *M. smegmatis*-laden filter cultures were quenched into 80% methanol precooled to −40 °C. Metabolites were extracted by mechanically lysing with specialized Lysing Matrix beads (MP Bio Lysing Matrix Tubes and FastPrep purification kits, MP Biomedicals LLC, USA) using a Mini-Bead Beater (BioSpec Products Inc.) with five cycles comprising 1 min homogenization and 1 min cooling on ice and lysates were clarified by centrifugation. Bacterial biomass was determined by measuring the residue protein content of individual samples. Bacterial biomasses (~2 mg whole lysate protein) were used for metabolomic analyses.

### High-performance liquid chromatography (HPLC) for Quantification of nucleotides bound to Rv2624c protein

Nucleotides bound to Rv2624c protein (or mutant Rv2624c proteins) were extracted by boiling purified Rv2624c for 5 min. Boiled samples were centrifuged and the supernatants were loaded onto an HPLC column (Mono Q HR5/5; GE Healthcare). Samples were eluted using a 1:1 mixture of Buffer A (0.04 M NaH_2_PO_4_, pH 5.5) and buffer B (1 M NaH_2_PO_4_, pH 5.5). The corresponding nucleotides were characterized based on retention time compared with ADP and ATP, and quantified by peak area calculations. Bovine serum albumin (BSA) was used as a negative control and experiments were performed in triplicate.

### Site-directed mutagenesis of Rv2624c

Mutation of specific amino acids in the ATP-binding conserved motif was incorporated in pQE-Rv2624c by mismatched PCR primers (listed below; bold and underline font indicated mutated nucleotides).

D17E-F: ATCATTGTTGGTATCGA**G**GGTTCGCACGCGGCG;

D17E-R: CGCCGCGTGCGAACC**C**TCGATACCAACAATGAT;

G109A-F: GAGATGATCTGCGTCG**C**CTCCGTGGGAATCGGG;

G109A-R: CCCGATTCCCACGGAG**G**CGACGCAGATCATCTC.

Individual PCR reactions were performed using either pQE-Rv2624c or pMV261-Rv2624c plasmids for Rv2624c protein expression and mycobacterial overexpression. Then, the PCR reaction mixtures were digested with DpnI (New England Biolabs, Ipswich, MA, USA). The mutant plasmid, pMV261-Rv2624cD17E and pMV261-Rv2624cG109A, was correspondingly transformed into *M. smegmatis* mc^2^155, and the mutant plasmid, pQE-Rv2624cD17E and pQE-Rv2624cG109A, was transformed into *E. coli* BL21 (DE3).

### *In vitro* growth kinetics

To determine the effect of Rv2624c overexpression and its mutations on growth, *M. smegmatis* strains harboring wild-type or mutant pMV261-Rv2624c (Rv2624cD17E and Rv2624cG109A) were grown in Middlebrook 7H9 medium containing 50 mg/L kanamycin. Log phase cultures (OD_600_ of 0.8–1.0) of mycobacterial strains were diluted into 7H9 medium to OD_600_ of 0.5. To generate a growth curve, cells were re-inoculated and OD_600_ was measured at the indicated times. To compare the size of colonies of different mycobacterial strains, cells were 10x serially diluted (1:10), spotted (5 μL) onto 7H10 medium supplemented with kanamycin (50 mg/L) and incubated at 37 °C. Photographs were taken after 3 days of incubation. The experiments were performed in triplicate.

### Macrophage killing assay

The macrophage killing assay was performed as previously described^33^. Briefly, human monocyte THP-1 cells (ATCC TIB-202) were differentiated by treatment with 100 μg/L phorbo-12-myristate-13-acetate (PMA; Sigma) for 24 h. Infection with various mycobacterial strains was carried out at a multiplicity of infection (MOI) of 10:1 for 4 h at 37 °C and 5% CO_2_. Unphagocytized bacilli were removed by extensively washing with PBS three times and the samples were incubated for 1 h with RPMI1640 containing 10 mg/L gentamycin to kill extracellular bacteria. The infected cells were lysed with PBS containing 0.05% Tween 20, and then diluted and plated on LB agar. The CFU number is indicated as the infected number of bacilli (T0). Next, infected THP-1 cells were incubated for 4 h, and then collected and diluted on LB agar. The corresponding post-infection CFUs were counted as the number of surviving bacilli (T1). The mc^2^155 strain with empty vector pMV261 was used as a negative control.

### RNA isolation, RNA-sequencing and data analysis

Bacterial cultures (OD_600_ of 2) of pMV261-Rv2624c/mc^2^155 and pMV261/mc^2^155 (negative control) were collected and total RNA was isolated using TRIzol reagent (Invitrogen, Carlsbad, CA, USA) according to the manufacturer’s instructions. Construction and sequencing of the above two strains was performed by BGI-Shenzhen (Shenzhen, China). Briefly, rRNA was removed from extracted RNA from the various mycboacterial strains using a Ribominus Transcriptome Isolation Kit (Thermo Fisher Scientific, Waltham, MA, USA). NEXTflex RNA Fragmentation Buffer (Bioo Scientific, Austin, Texas, USA) was added to cleave mRNA into short fragments. Using these short fragments as templates, random hexamer primers were used to synthesize the first-strand cDNA. Second-strand cDNA was synthesized using buffer, dATPs, dGTPs, dCTPs, dUTPs, RNase H and DNA polymerase I. Short fragments were purified with a QIAQuick PCR extraction kit (Qiagen) and resolved by electrophoresis for end reparation and addition of poly(A). After ligating the synthetic short fragments to sequencing adapters, UNG enzyme was used to degrade second-strand cDNA, and the product was purified with a MiniElute PCR Purification Kit (Qiagen). The library was sequenced using an Illumina HiSeq2000. Clean reads were mapped to the reference genome and gene sequences using SOAP2. Mismatches of no more than five bases were allowed in the alignment. Gene coverage is the percentage of a gene covered by reads and is equal to the ratio of the number of bases in a gene covered by unique mapping reads to the number of total bases in that gene. The RPKM method (reads per kilobase per million reads)^34^ was used to calculate gene expression. The algorithm for identifying differentially expressed genes between two samples was developed by BGI Shenzhen, and the false discovery rate (FDR) control was used for correct *p* values. In this study, differential expression was indicated when the false discovery rate was ≤0.001 and the ratio was larger than 2. Analysis of differentially expressed genes was further carried out using the KEGG Pathway analysis^24^.

### Statistical analysis

GraphPad Prism 6.0c was used to perform statistical analysis. Significant differences in the data were calculated using *t*-tests. *p* < 0.05 indicated significant differences.

### LC-MS/MS Analysis of Arginine

An Agilent 1260 HPLC coupled with an AB SCIEX QTRAP 4500 system (AB SCIEX, Foster, CA, USA) was used for LC-MS/MS analysis. The compounds were separated on a Synergi 4 μm Hydro-RP 80 ALC column (2 × 150 mm) at a column temperature of 25 °C. The elution solvent system composed of ultra-pure water (solvent A) and methanol (solvent B). The injection volume of the autosampler was 3 μL. The gradient elution program was applied at a flow rate of 0.4 mL/min as follows: 0 min, 2% B; 5 min, 2% B; 40 min, 100% B; 50 min, 100% B; 52 min, 5% B; 60 min, 5% B.

Mass spectrometry was carried out using electrospray ionization (ESI). MS analyses were conducted in positive-ion mode. The operating parameters were optimized as follows: curtain gas (CUR): 20.0; collision gas (CAD): medium; IonSpray voltage (IS): 5500 V; temperature: 500 °C; ion source gas 1 (GS1): 60.0; ion source gas 2 (GS2): 60.0; declustering potential (DP): 50.0; entrance potential (EP): 10; collision energy (CE): 20; collision cell exit potential (CXE): 13.0; Ion detection was performed in multiple reaction monitoring (MRM) mode and the scanning time for every ion pair was 100 ms. MRM transitions monitored for arginine were 175 → 116.

## Additional Information

**How to cite this article**: Jia, Q. *et al.* Universal stress protein Rv2624c alters abundance of arginine and enhances intracellular survival by ATP binding in mycobacteria. *Sci. Rep.* **6**, 35462; doi: 10.1038/srep35462 (2016).

## Supplementary Material

Supplementary Information

## Figures and Tables

**Table 1:** Fold changes of genes differentially expressed.

Gene Name	Gene Product	log2 Ratio (pMV261-Rv2624c/pMV261)	P-value	FDR
Arginine and proline metabolism				
MSMEI_4735	alpha/beta hydrolase fold protein;	2.08729664	1.85E-05	7.24E-05
aldA	aldehyde dehydrogenase;	1.790903637	7.18E-07	3.42E-06
MSMEI_5246	K transporting ATPase KdpC subunit;	1.619791721	4.22E-05	0.000155477
argB	acetylglutamate kinase;	1.588936971	1.08E-112	7.83E-111
MSMEI_3474	nitrilase/cyanide hydratase and apolipoprotein N-acyltransferase;	1.541289747	1.41E-23	1.85E-22
MSMEI_6319	aldehyde dehydrogenase;	1.524828777	1.89E-07	9.60E-07
MSMEI_3879	5-oxoprolinase;	1.502683418	6.55E-13	5.31E-12
MSMEI_0270	aminotransferase class-III;	1.449866719	6.83E-05	0.000242346
MSMEI_2911	amidase;	1.429108159	3.54E-06	1.53E-05
argJ	Arginine biosynthesis bifunctional protein argJ;	1.412545596	1.55E-131	1.31E-129
argD	acetylornithine aminotransferase;	1.39097303	1.56E-137	1.44E-135
MSMEI_2053	acetylornithine deacetylase/Succinyl-diaminopimelate desuccinylase-like protein;	1.362958654	2.43E-11	1.76E-10
MSMEI_0432	agmatine deiminase;	1.243415842	0.000305502	0.000956325
MSMEI_0187	RpiR-family transcriptional regulator;	1.172332744	0.000101264	0.000348923
aldA	aldehyde dehydrogenase AldA;	1.145012138	1.47E-05	5.85E-05
amiD	amidase;	1.116105106	2.68E-137	2.44E-135
MSMEI_1056	Asp-tRNAAsn/Glu-tRNAGln amidotransferase A subunit-like amidase;	1.084415112	8.54E-60	2.94E-58
argC	N-acetyl-gamma-glutamyl-phosphate reductase;	1.065976916	4.85E-48	1.28E-46
aldA	NAD-dependent aldehyde dehydrogenase AldA;	1.002407743	7.59E-05	0.000267238
Histidine metabolism				
purU	formyltetrahydrofolate deformylase;	0.015303426	0.817688	0.861173926
MSMEI_1150	formiminoglutamate deiminase;	1.550910305	1.54E-86	8.22E-85
MSMEI_1151	Imidazolonepropionase;	1.544346856	7.79E-56	2.44E-54
MSMEI_6319	aldehyde dehydrogenase;	1.524828777	1.89E-07	9.60E-07
MSMEI_2769	nitrilotriacetate monooxygenase;	1.49877632	5.41E-07	2.61E-06
MSMEI_3211	Adenylate/guanylate cyclase/hydrolase, alpha/beta fold family;	1.481575579	5.06E-25	7.20E-24
MSMEI_3620	catalase;	1.379856998	0	0
MSMEI_2508	alpha/beta hydrolase fold-3;	1.37437652	2.33E-09	1.43E-08
MSMEI_1152	histidine ammonia-lyase;	1.333291143	4.48E-64	1.73E-62
MSMEI_6690	hypothetical protein;	1.297863626	3.89E-05	0.000144317
MSMEI_1148	urocanate hydratase;	1.198212357	0	0
MSMEI_2768	nitrilotriacetate monooxygenase;	1.162704043	1.29E-05	5.19E-05
aldA	aldehyde dehydrogenase AldA;	1.145012138	1.47E-05	5.85E-05
MSMEI_4521	phthalate 4,5-dioxygenase;	1.061120518	1.08E-20	1.31E-19
aldA	NAD-dependent aldehyde dehydrogenase AldA;	1.002407743	7.59E-05	0.000267238
