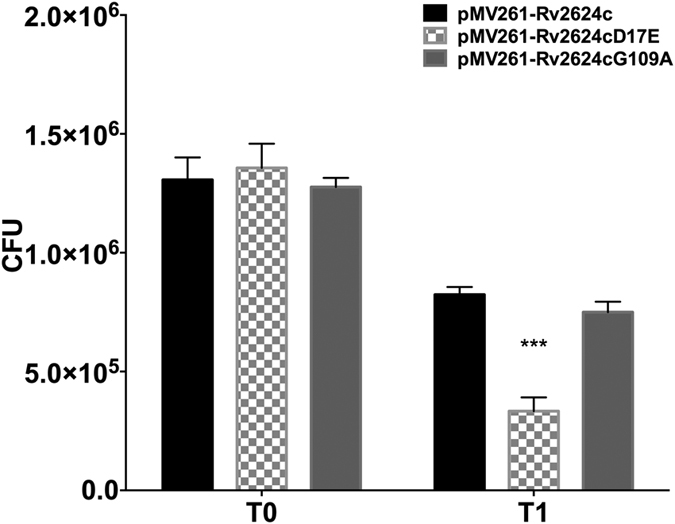# Corrigendum: Universal stress protein Rv2624c alters abundance of arginine and enhances intracellular survival by ATP binding in mycobacteria

**DOI:** 10.1038/srep44966

**Published:** 2017-03-22

**Authors:** Qiong Jia, Xinling Hu, Dawei Shi, Yan Zhang, Meihao Sun, Jianwei Wang, Kaixia Mi, Guofeng Zhu

Scientific Reports 6: Article number: 35462; 10.1038/srep35462 published online: 11 20 2016; updated: 03 22 2017.

This Article contains errors in Figure 5B where the CFU values for T0 and T1 are incorrect. The correct Figure 5B appears below as Figure 1.

## Figures and Tables

**Figure 1:**