# Supplementary material for: Universal stress protein Rv2624c alters abundance of arginine and enhances intracellular survival by ATP binding in mycobacteria
Source: Sci Rep. 2016 Oct 20;6:35462. doi: 10.1038/srep35462 (PMC5071874; doi:10.1038/srep35462)
Supplement: Supplementary Information [file srep35462-s1.doc]

**Universal stress protein Rv2624c alters abundance of arginine and enhances intracellular survival by ATP binding in mycobacteria**

Qiong Jia^1#^, Xinling Hu^2, 3#^, Dawei Shi^4^, Yan Zhang^5^, Meihao Sun^6^, Jianwei Wang^1^, Kaixia Mi^2*^, Guofeng Zhu^7*^

^1^ MOH Key Laboratory of Systems Biology of Pathogens, Institute of Pathogen Biology, Chinese Academy of Medical Sciences and Peking Union Medical College, Beijing 100730, China

^2^ CAS Key Laboratory of Pathogenic Microbiology and Immunology, Institute of Microbiology, CAS, Beijing 100101, China

^3^ The Research Institute of Forestry, Chinese Academy of Forestry, Beijing 100091, China

^4^ Key Laboratory of the Ministry of Health for Research on Quality and Standardization of Biotech Products, National Institutes for Food and Drug Control, Beijing 100050, China

^5^ Zhangjiakou Center for Adverse Drug Reaction and Drug Abuse, Hebei 075000, China

^6^ College of Chemistry and Life Sciences, Zhejiang Normal University, Zhejiang 321004, China

^7^ Shanghai Municipal Center for Disease Control & Prevention, Shanghai 200336, China

^#^ These authors have contributed equally to this work.

^*^ Corresponding authors:

Kaixia Mi

Institute of Microbiology, CAS, Beijing 100101, China

Phone: 86-10-64806082, Fax: 86-10-64807462

Email: mik@im.ac.cn

Guofeng Zhu, Ph.D.

Shanghai Municipal Center for Disease Control & Prevention, Shanghai 200336, China

Phone: 86-21-62700323, Fax: 86-21-62700323

Email: [zhugf@yahoo.com](mailto:zhugf@yahoo.com)

**Supplementary Figure**


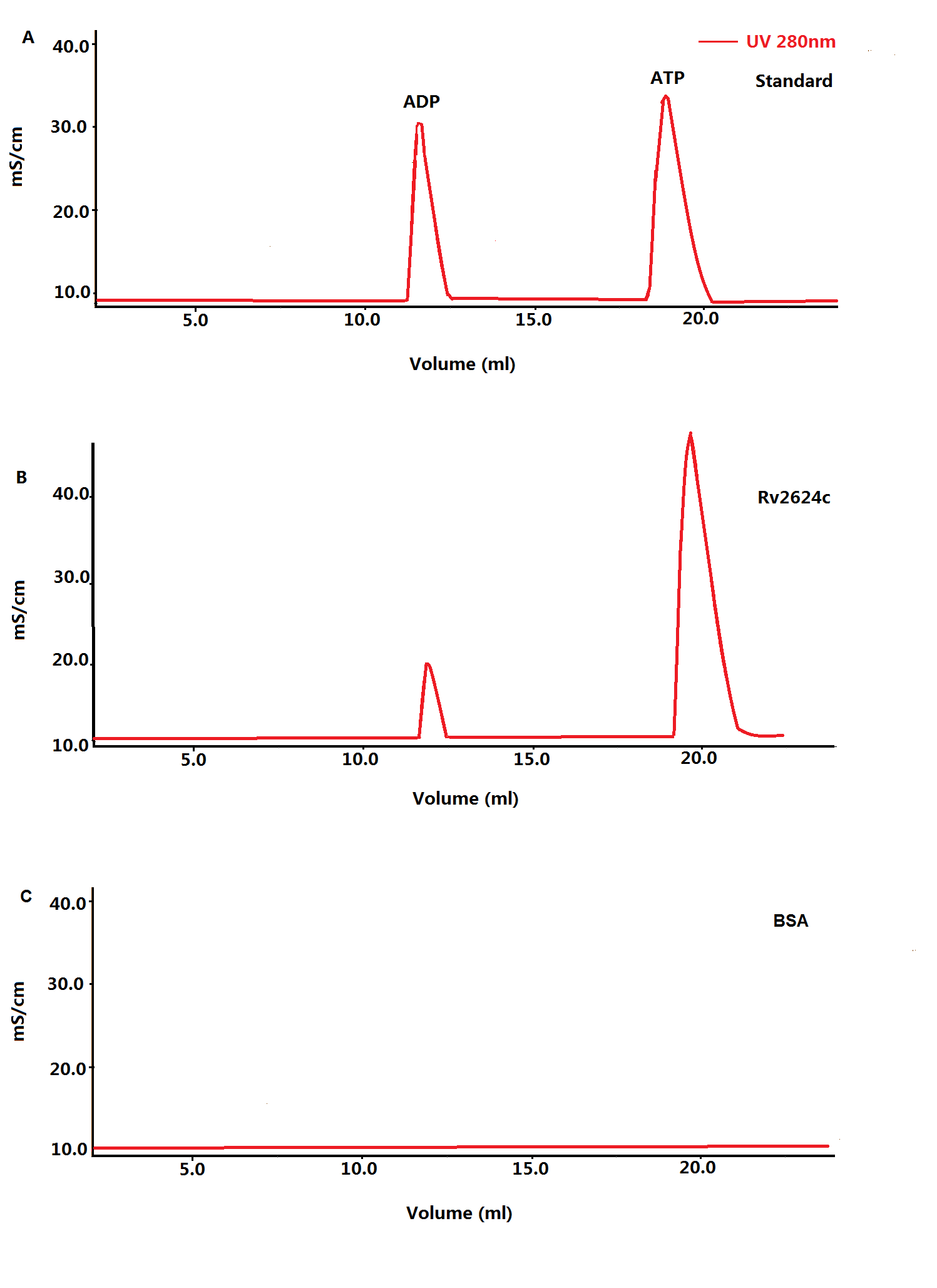


**Supplementary Figure 1 Rv2624c is a nucleotide-binding universal stress protein**

High-performance liquid chromatography (HPLC) of nucleotides endogenously bound to purified Rv2624c. The upper panel is the profile of standard ADP and ATP. The amount of the corresponding bound nucleotides extracted from Rv2624c proteins was calculated based on specific retention times on the Superdex 200 10/300 GL column (middle panel). The corresponding bound nucleotides extracted from BSA, a negative control (lower panel).

**Supplementary Table**

**Supplementary Table 1 Oligonucleotide primers used in this study**

| **Name** | **5’-3’** | **Purpose** |
| --- | --- | --- |
| Rv2624c-F | TTTTGGATCCATGTCTGGGAGAGGAGAGCCGACG | pMV261- Rv2624c |
| Rv2624c-R | TTTTAAGCTTCAGCGGCGAACGACAAGCACCGAA |
| D17E-F | ATCATTGTTGGTATCGA**G**GGTTCGCACGCGGCG | pMV261-Rv2624cD17E |
| D17E-R | CGCCGCGTGCGAACC**C**TCGATACCAACAATGAT |
| G109A-F | GAGATGATCTGCGTCG**C**CTCCGTGGGAATCGGG | pMV261-Rv2624cG109A |
| G109A-R | CCCGATTCCCACGGAG**G**CGACGCAGATCATCTC |
